# Supplementary figures and images for: Deep Learning Enables Individual Xenograft Cell Classification in Histological Images by Analysis of Contextual Features
Source: J Mammary Gland Biol Neoplasia. 2021 May 17;26(2):101–12. doi: 10.1007/s10911-021-09485-4 (PMC8236058; doi:10.1007/s10911-021-09485-4)

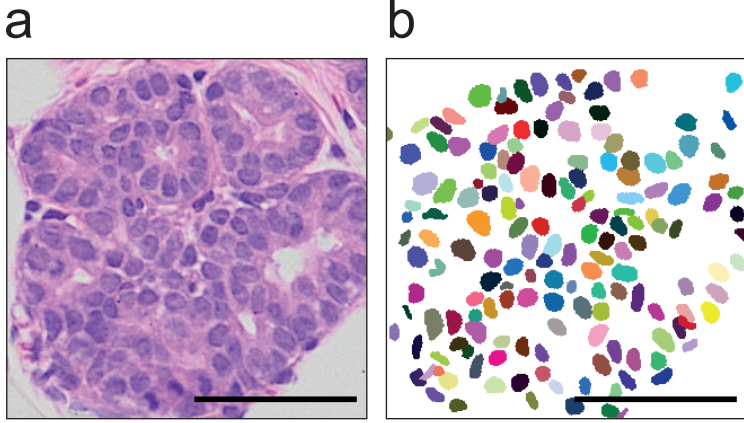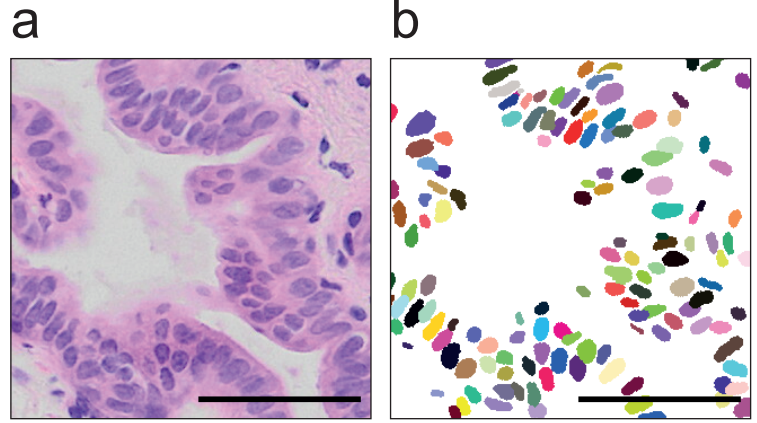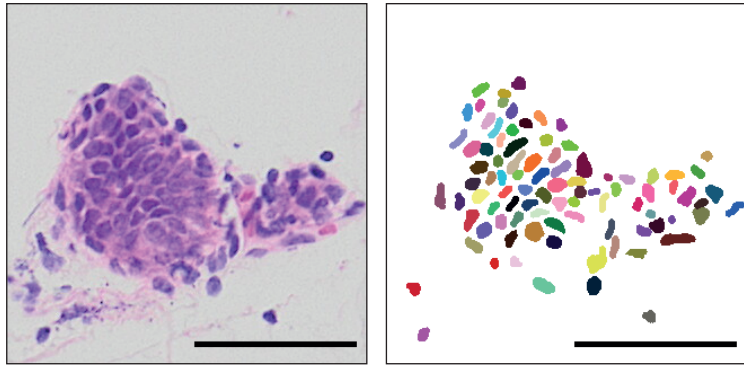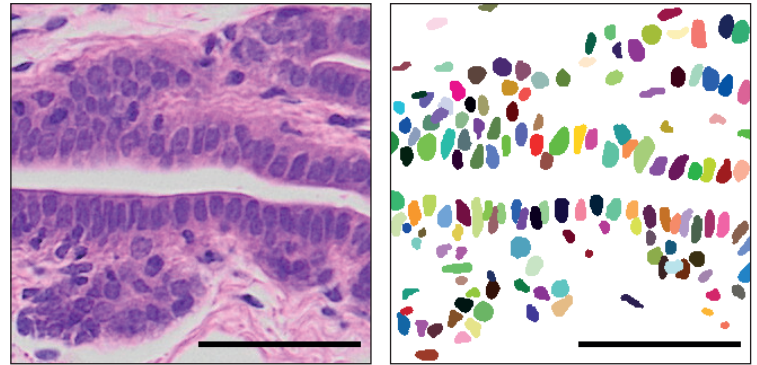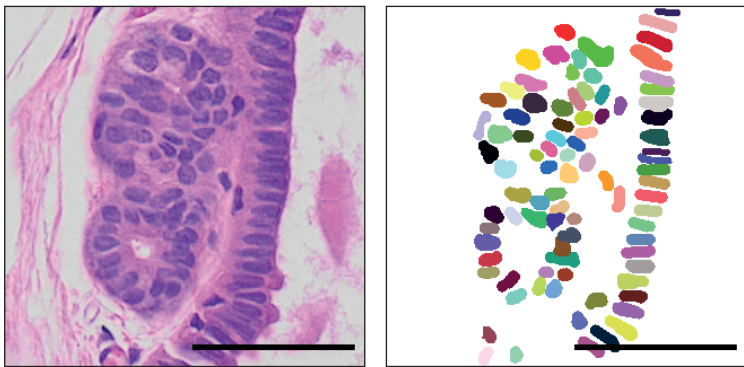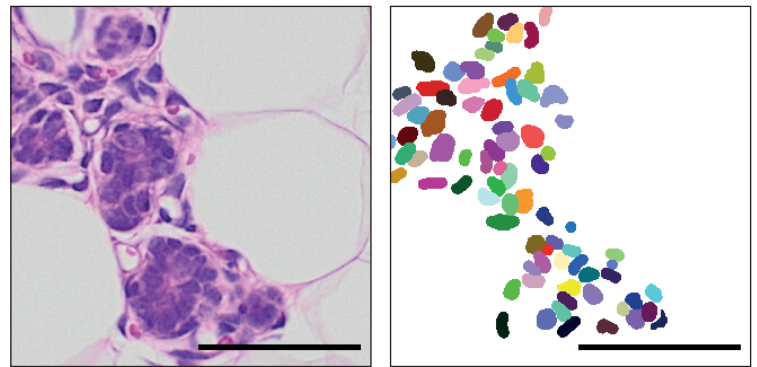

Supplement: Supplementary file 1 — (PDF 3.15 MB) [file 10911_2021_9485_MOESM1_ESM.pdf]

a

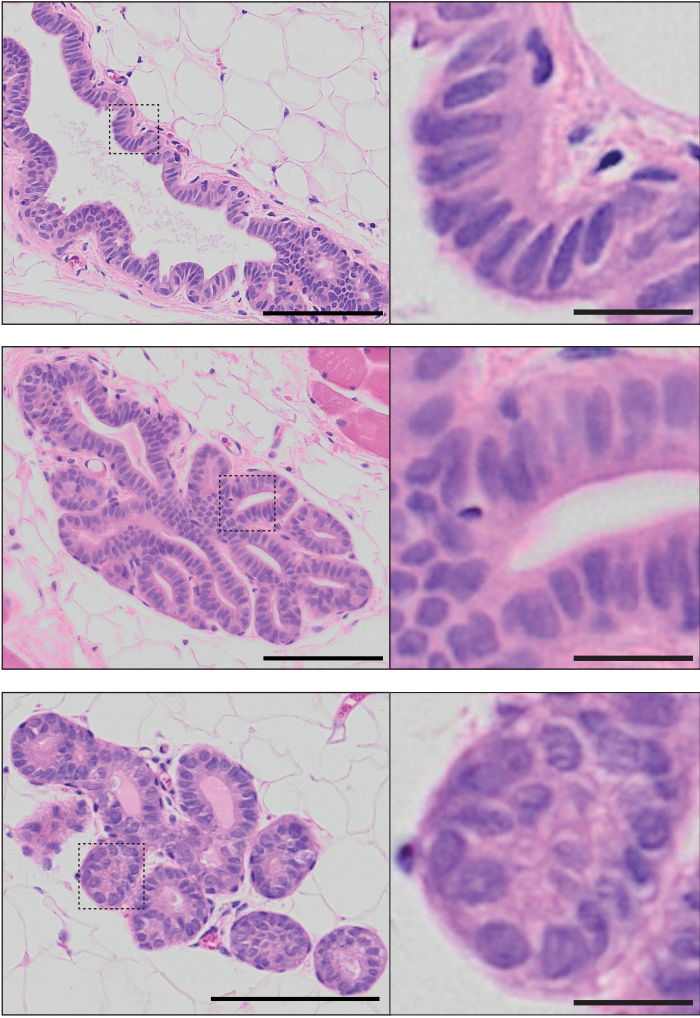

b

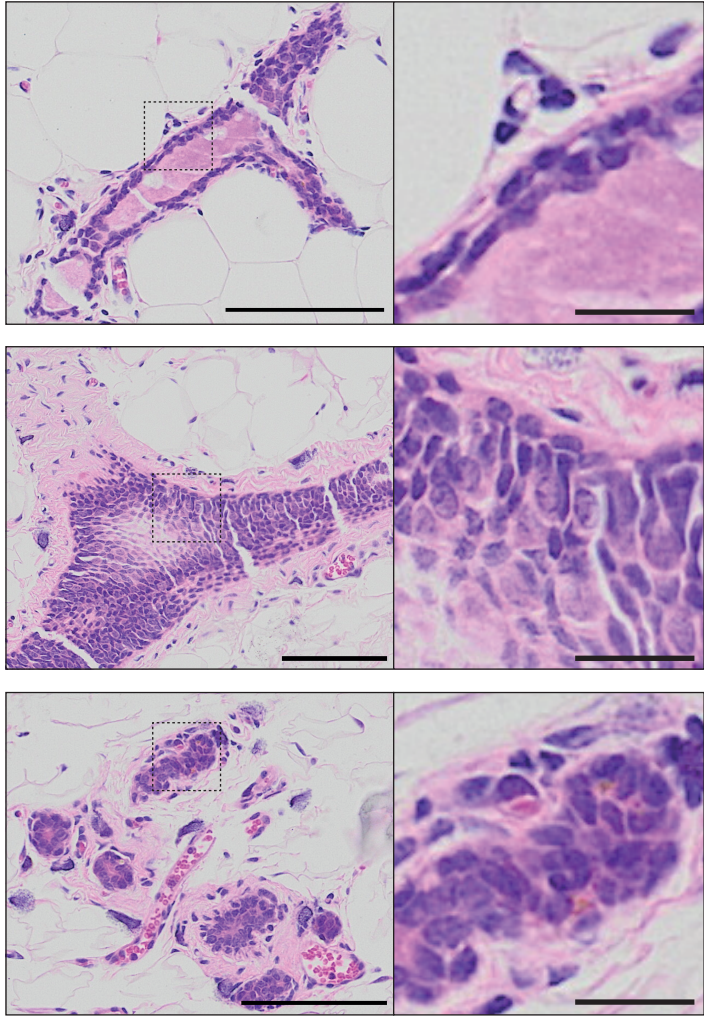

Supplement: Supplementary file 2 — (PDF 16.2 MB) [file 10911_2021_9485_MOESM2_ESM.pdf]

a

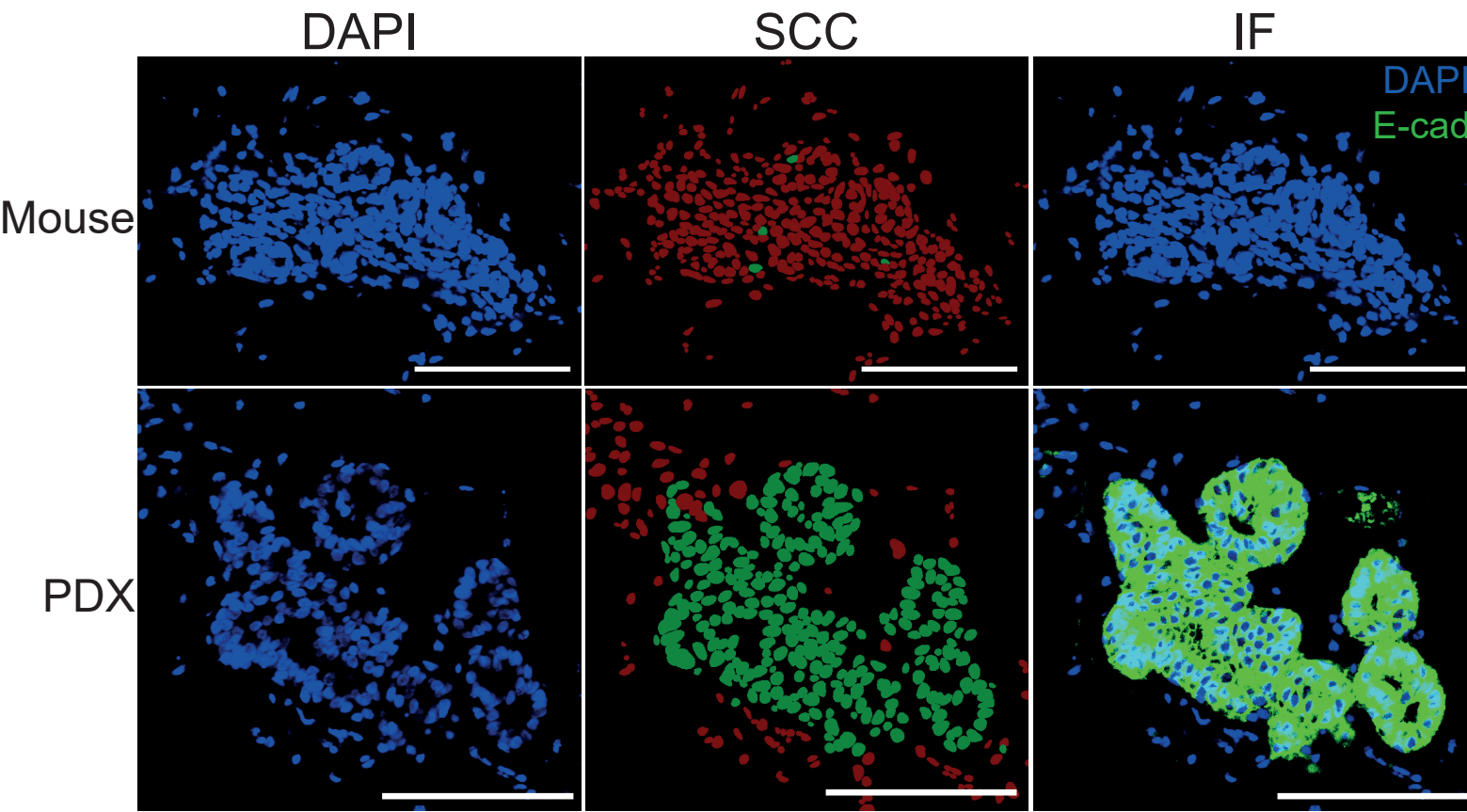

Supplement: Supplementary file 3 — (PDF 1.8 MB) [file 10911_2021_9485_MOESM3_ESM.pdf]

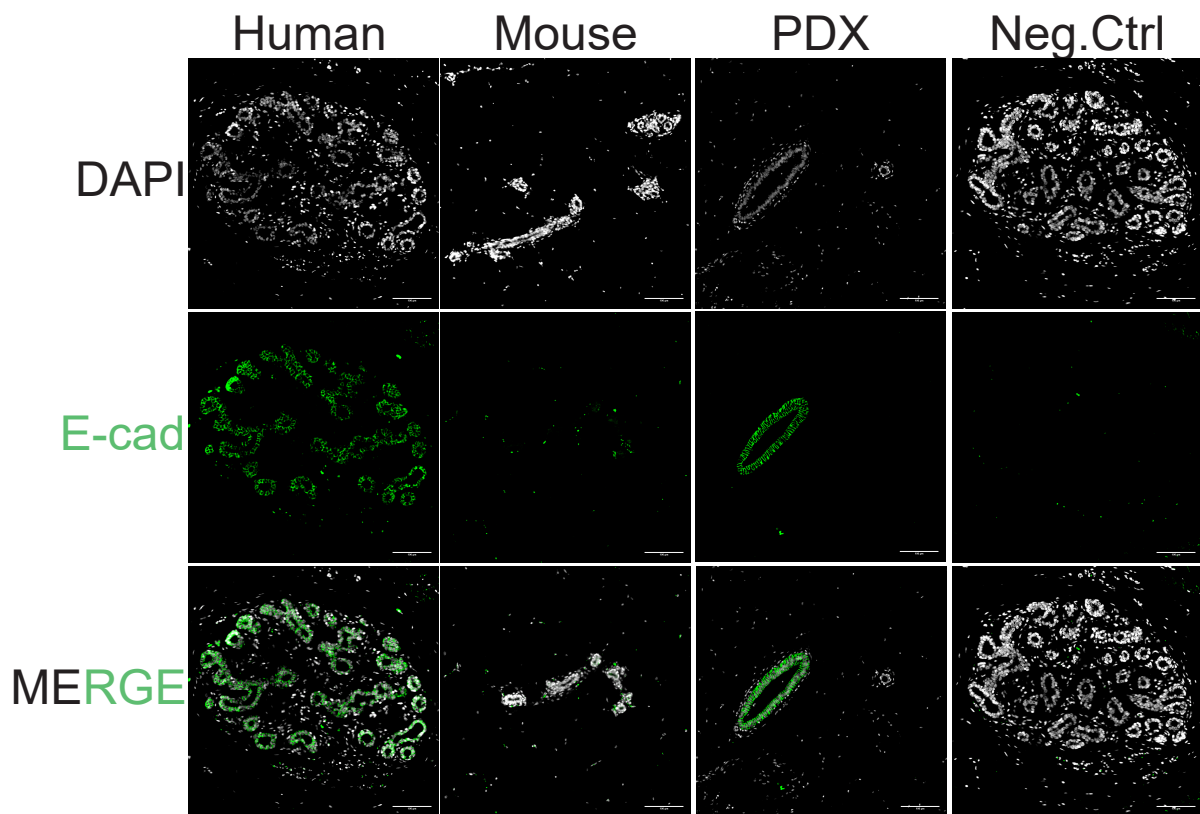

Supplement: Supplementary file 4 — (PDF 7.7 MB) [file 10911_2021_9485_MOESM4_ESM.pdf]

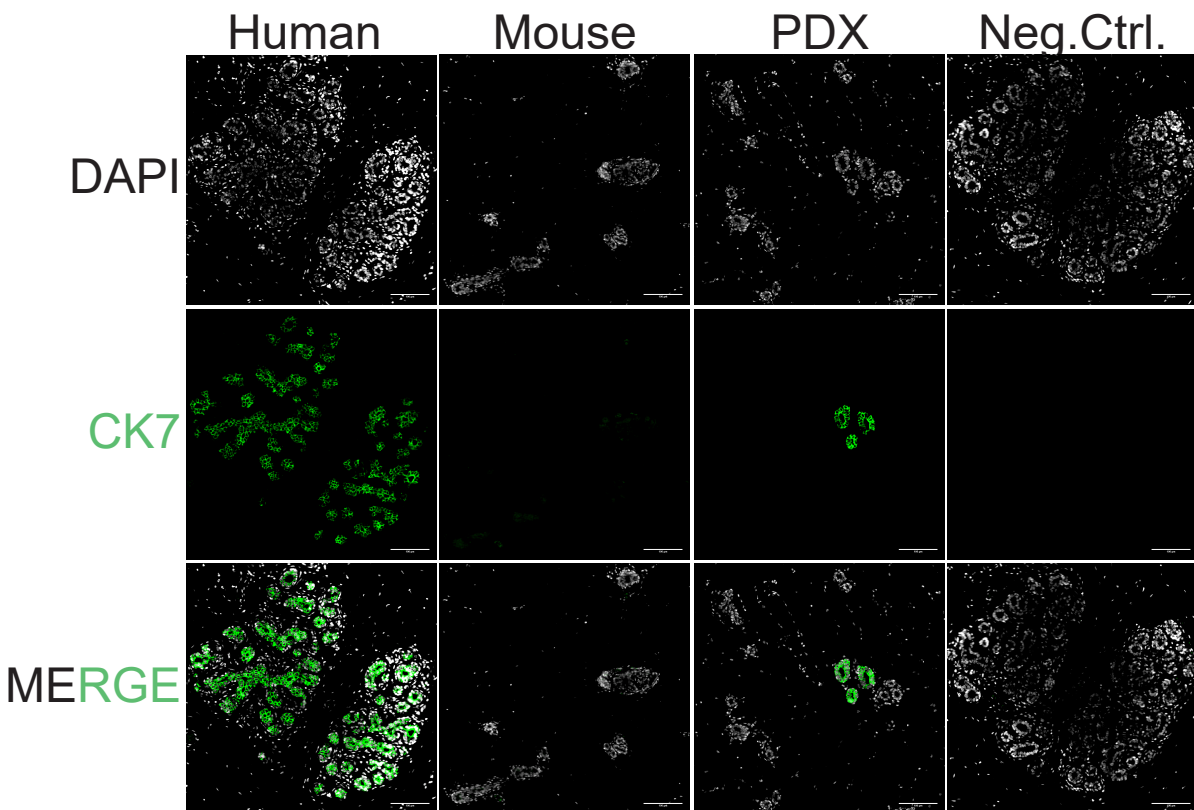

Supplement: Supplementary file 5 — (PDF 8.6 MB) [file 10911_2021_9485_MOESM5_ESM.pdf]
