# Supplementary material for: Deep Learning Enables Individual Xenograft Cell Classification in Histological Images by Analysis of Contextual Features
Source: J Mammary Gland Biol Neoplasia. 2021 May 17;26(2):101–12. doi: 10.1007/s10911-021-09485-4 (PMC8236058; doi:10.1007/s10911-021-09485-4)
Supplement: Supplementary file 7 — (PDF 0.96 MB) [file 10911_2021_9485_MOESM7_ESM.pdf]

Supplementary Material

Supp. Note 1 — Detection Metrics Details

To quantify the accuracy of our detection results, we first compute for every image a bipartite matching between ground truth objects and predictions, where a ground truth object O_{GT} and a predicted object O_P are considered a match if their intersection over union IoU meets the following condition:

$$IoU = \frac{O_{GT} \cap O_P}{O_{GT} \cup O_P} \geq 0.5$$

Matched predictions are then considered as true positives (TP), unmatched ground truth objects as false negatives (FN), and unmatched predictions as false positives (FP). We finally count the number of TP, FP, and FN across all images and compute the accuracy (average precision) AP, precision P, sensitivity (recall) S, and F1 score F_1 with the following computations:

$$\begin{aligned} AP &= \frac{TP}{TP + FN + FP} \\ P &= \frac{TP}{TP + FP} \\ S &= \frac{TP}{TP + FN} \\ F_1 &= \frac{2TP}{2TP + FP + FN} \end{aligned}$$
